# Efficient hydrolysis of raw starch and ethanol fermentation: a novel raw starch-digesting glucoamylase from *Penicillium oxalicum*

**DOI:** 10.1186/s13068-016-0636-5

**Published:** 2016-10-18

**Authors:** Qiang-Sheng Xu, Yu-Si Yan, Jia-Xun Feng

**Affiliations:** State Key Laboratory for Conservation and Utilization of Subtropical Agro-bioresources, Guangxi Key Laboratory of Subtropical Bioresources Conservation and Utilization, Key Laboratory of Ministry of Education for Microbial and Plant Genetic Engineering, College of Life Science and Technology, Guangxi University, 100 Daxue Road, Nanning, 530004 Guangxi People’s Republic of China

**Keywords:** *Penicillium oxalicum*, Raw starch-digesting glucoamylase, Gene cloning and expression, Corn starch, Cassava starch, Raw starch hydrolysis, Simultaneous saccharification and fermentation, Ethanol

## Abstract

**Background:**

Starch is a very abundant and renewable carbohydrate and is an important feedstock for industrial applications. The conventional starch liquefaction and saccharification processes are energy-intensive, complicated, and not environmentally friendly. Raw starch-digesting glucoamylases are capable of directly hydrolyzing raw starch to glucose at low temperatures, which significantly simplifies processing and reduces the cost of producing starch-based products.

**Results:**

A novel raw starch-digesting glucoamylase PoGA15A with high enzymatic activity was purified from *Penicillium oxalicum* GXU20 and biochemically characterized. The PoGA15A enzyme had a molecular weight of 75.4 kDa, and was most active at pH 4.5 and 65 °C. The enzyme showed remarkably broad pH stability (pH 2.0–10.5) and substrate specificity, and was able to degrade various types of raw starches at 40 °C. Its adsorption ability for different raw starches was consistent with its degrading capacities for the corresponding substrate. The cDNA encoding the enzyme was cloned and heterologously expressed in *Pichia pastoris*. The recombinant enzyme could quickly and efficiently hydrolyze different concentrations of raw corn and cassava flours (50, 100, and 150 g/L) with the addition of α-amylase at 40 °C. Furthermore, when used in the simultaneous saccharification and fermentation of 150 g/L raw flours to ethanol with the addition of α-amylase, the ethanol yield reached 61.0 g/L with a high fermentation efficiency of 95.1 % after 48 h when raw corn flour was used as the substrate. An ethanol yield of 57.0 g/L and 93.5 % of fermentation efficiency were achieved with raw cassava flour after 36 h. In addition, the starch-binding domain deletion analysis revealed that SBD plays a very important role in raw starch hydrolysis by the enzyme PoGA15A.

**Conclusions:**

A novel raw starch-digesting glucoamylase from *P. oxalicum*, with high enzymatic activity, was biochemically, molecularly, and genetically identified. Its efficient hydrolysis of raw starches and its high efficiency during the direct conversion of raw corn and cassava flours via simultaneous saccharification and fermentation to ethanol suggests that the enzyme has a number of potential applications in industrial starch processing and starch-based ethanol production.

**Electronic supplementary material:**

The online version of this article (doi:10.1186/s13068-016-0636-5) contains supplementary material, which is available to authorized users.

## Background

Starch is one of the most abundant renewable carbohydrate reserves in a large variety of higher plants, such as cereals, tuberous plants, and legumes. It is a polymer of glucose, and mainly consists of amylose and amylopectin. Amylose is a mostly linear molecule containing α-d-glucosyl units that are essentially linked by α-1,4-glycosidic bonds, whereas amylopectin is a highly branched structure composed of large polymers of α-1, 4-glycosidic bonds linked α-d-glucosyl units with α-1, 6-linked side chains [1].

Starch is the second most important and abundant source of carbon and energy in plants, and thus has large market demand and many applications in industry. It can be used to produce many valuable food products in the food processing industry, such as maltose, glucose, fructose, glucose–fructose syrups, organic acids, amino acids, etc. [2]. Furthermore, starch is also an important feedstock in the fermentation industry and is widely saccharified and fermented to produce ethanol, which can be used as a basis for beverages or as an alternative biofuel [3].

Alpha-amylase (EC 3.2.1.1) is an endo-acting enzyme that can catalyze the hydrolysis of α-1, 4-glycosidic linkages and some branched α-1, 6-glycosidic linkages from the inner chains of starch. This primarily leads to the release of maltose, smaller oligosaccharides, and dextrin as the main products [4]. In contrast, glucoamylase (EC 3.2.1.3) is an exo-acting enzyme that mainly hydrolyzes α-1, 4-glycosidic linkages from the non-reducing ends of starch chains, which leads to the production of glucose [5].

Conventionally, the way to convert starch to smaller oligosaccharides and glucose in industry includes energy-intensive liquefaction and saccharification, which mainly involve α-amylase and glucoamylase hydrolysis under high temperature conditions. In the primary liquefaction step, starch is first gelatinized and then liquefied to dextrin and small molecules by a thermophilic α-amylase from a bacterium at high temperature (95–105 °C) and at pH 6.0–6.5. In the following saccharification step, the liquefied starch slurry is cooled and the pH adjusted to 4.0–4.5. Glucoamylase from a fungus is added to further hydrolyze the oligosaccharides to glucose at the lower temperatures of 60–65 °C [6]. Starch enzymatic hydrolysis at high temperature requires large energy inputs and extra equipment, which results in an increased cost of production for starch-derived commodities [7]. Regardless of whether it is glucose–fructose syrup or ethanol that is produced from the starch feedstock, starch first needs to be hydrolyzed and saccharified into monomeric glucose. Thus, direct and efficient hydrolysis of raw starch under low temperature would save energy and be more cost-effective, indicating that it is very important to identify an enzyme that is capable of effectively digesting raw granular starch under low temperature conditions [8, 9].

Raw starch-digesting enzymes are enzymes that are able to directly hydrolyze raw starch granules below the starch gelatinization temperature. This significantly reduces the large energy inputs required and simplifies the manufacturing process for starch-based products because there would be no need for liquefaction and saccharification [10]. The energy input for starch liquefaction and saccharification represents about 30–40 % of the total energy used during starch-based ethanol production [11]. Robertson et al. [12] reviewed the raw starch-digesting enzymes and estimated that efficient raw starch-digesting enzyme applied in ethanol production could lead to a significant energy usage reduction of about 10–20 % of the fuel value of the total ethanol product.

Only approximately 10 % of amylolytic enzymes are able to digest raw starch because of the particle size and the densely compacted polycrystalline architecture of natural starch granules. Several types of raw starch-digesting enzymes, such as α-amylase, glucoamylase, and β-amylase, have been isolated from plants, animals, and microorganisms [10, 13]. A large number of microorganisms, including fungi and bacteria, have been reported to produce raw starch-digesting enzymes, such as α-amylases, but only a few studies about raw starch-digesting glucoamylase (RSDG) have been published [10]. RSDG can hydrolyze raw starch directly to produce glucose as the sole product in a single step, which would simplify starch processing and reduce energy consumption during the industrial production of starch-based products.

The RSDGs are mainly produced by fungi or yeasts, such as *Aspergillus niger* [14, 15], *Aspergillus oryza*e [16], *Aspergillus awamori* [17], *Corticium rolfsii* [18], *Penicillium* sp. [19, 20], and *Aureobasidium pullulans* [21]. RSDGs have previously been purified from the crude extract and biochemically characterized. Flor et al. [17] purified and characterized an RSDG (GAO) with a large molecular weight of 250 kDa from *A. awamori* var. *kawachi*. Two glucoamylases (GA1 and GA2) purified from *A. niger* showed different raw starch-degrading abilities and GA2 was approximately 6–13 times more active against raw starches than GA1 [22]. Nagasaka also reported that three out of five forms of purified glucoamylases (G1, G2, and G3) had similar enzyme characteristics and were able to hydrolyze cereal raw starch, but had a poor performance against root raw starch [18]. A thermostable RSDG purified from *Thermomucor indicae*-*seudaticae* was a glycoprotein and acted optimally at pH 7.0 and 60 °C [23]. An extracellular RSDG purified from the marine yeast *Aureobasidium pullulans* showed optimum activity at 60 °C and pH 4.5, but could only digest raw potato starch even though it possesses various raw starch adsorption abilities [24]. These reported enzymes are capable of raw starch digestion, but their properties vary depending on their source. However, there have been few reports of an RSDG that has a high enzyme activity and broad substrate specificity at low temperature, which would reduce the cost of its application in the hydrolysis of different raw starches and simultaneous saccharification and fermentation of raw starch to ethanol. Therefore, it is important to identify and characterize a new RSDG with high enzymatic activity and broad substrate specificity, and apply it to raw starch hydrolysis and ethanol fermentation.

In previous studies, we reported that a crude enzyme preparation from *Penicillium* sp. GXU20 displayed high enzyme activity towards various raw starches. It could effectively hydrolyze 150 g/L of raw cassava flour into glucose as the main product, and a high ethanol yield (53.3 g/L) and fermentation efficiency (92 %) were achieved by simultaneous saccharification and fermentation (SSF) using the crude enzyme after 48 h at 40 °C [20].

In this study, a novel RSDG was purified from *Penicillium* sp. GXU20 and biochemically characterized. The gene coding for the enzyme was cloned and heterologously expressed in *Pichia pastoris*. Then the recombinant enzyme was used to study the hydrolysis of raw corn and cassava flours and the simultaneous saccharification and fermentation of the raw flours to ethanol with the addition of α-amylase. This produced a rapid and efficient hydrolysis rate, and a high fermentation efficiency.

## Results and discussion

### Purification and identification of a novel raw starch-digesting enzyme from *P. oxalicum* GXU20

**Fig. 1:**
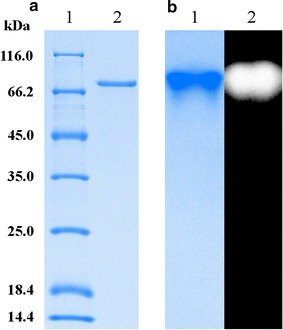
SDS-PAGE and native-PAGE analysis of the purified raw starch-digesting enzyme. **a** SDS-PAGE analysis of the purified raw starch-digesting enzyme. *Lane 1* protein molecular weight marker; *lane 2* the purified raw starch-digesting enzyme. **b** Native-PAGE of the purified enzyme. *Lane 1* the purified protein stained by Coomassie Brilliant Blue R-250; *lane 2* amylase activity visualized by KI/I_2_ solution

During enzyme purification, the ability to hydrolyze raw starch was monitored when raw cassava starch was used as a substrate. After ethanol precipitation and two chromatographic separation steps, a raw starch-digesting enzyme was purified to homogeneity on SDS-PAGE and native-PAGE (Fig. 1). The results of the enzyme purification are summarized in Table 1. Ethanol precipitation was a very effective approach that separated the raw starch-digesting enzyme from a large number of proteins in the supernatant and produced a protein yield of 53.51 %. The 60.61-fold purification was achieved with a yield of 11.60 % after two successive chromatography steps (Hiprep 16/10 Phenyl FF and Source 15S 4.6/100 PE).

**Table 1:** Purification of a raw starch-digesting enzyme from *P*. *oxalicum* GXU20

Purification step	Total activity (U)	Total protein (mg)	Specific activity (U/mg)	Yield (%)	Fold purification
Crude supernatant	936.81	5233.64	0.18	100.00	1.00
Ethanol precipitation	501.28	418.05	1.20	53.51	6.67
Hiprep 16/10 Phenyl FF	314.44	127.28	2.47	33.56	13.72
Source 15S 4.6/100 PE	108.68	9.96	10.91	11.60	60.61

Trypsin-digested peptide fragments of the purified enzyme were analyzed by liquid chromatography–tandem mass spectrometry (LC/MS/MS) [25, 26]. Three internal peptide fragments of the purified enzyme matched the peptide sequences ALANHKVYTDSFR, IGSITITSTSLAFFK, and YPEDSYYGGNPWFLSNLAAAEQLYDAIYQWNK, which are derived from putative glucoamylases belonging to glycosyl hydrolase family 15, from *P. marneffei* ATCC 18224 (XP_002149243), and *P. oxalicum* 114-2 (EPS34453).

**Fig. 2:**
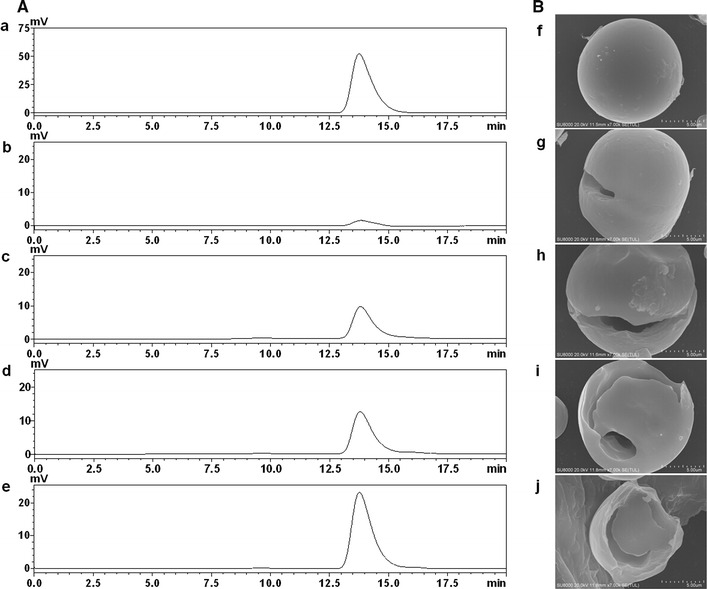
HPLC chromatograms of raw cassava starch hydrolysates and SEM analysis of the granules treated by the glucoamylase PoGA15A. **A** HPLC analysis of the reaction mixture produced by the purified enzyme. *a* Glucose standard, and products from raw cassava starch hydrolyzed by the enzyme after 0.5 h (*b*), 2 h (*c*), 4 h (*d*), and 8 h (*e*). **B** Scanning electron micrographs of raw cassava starch granules digested by the purified enzyme. *f* An untreated raw cassava starch granule, and raw cassava starch granules treated after enzyme hydrolysis for 0.5 h (*g*), 2 h (*h*), 4 h (*i*), and 8 h (*j*)

The hydrolysis products from raw cassava starch by the purified enzyme were separated and identified by HPLC (Fig. 2A). When the purified enzyme was incubated with 2 % raw cassava flour at 40 °C and pH 4.5, only a small amount of glucose was produced during the early stage of the hydrolysis reaction (30 min). As the reaction proceeded, glucose remained the sole hydrolysis product (no maltose or maltotriose was detected) and its concentration gradually increased. These results clearly demonstrated that the purified enzyme was an exo-acting amylase that cleaves glycosidic bonds successively to release glucose from the non-reducing ends of starch chains, thereby indicating that the purified amylase from *P. oxalicum* GXU20 was a glucoamylase.

The data from the mass spectrometry analysis of the purified enzyme and the hydrolysis products detected by HPLC confirmed that the purified raw starch-digesting enzyme from *P. oxalicum* GXU20 was a glucoamylase. The purified enzyme was named PoGA15A.

The molecular weight of the purified enzyme PoGA15A was estimated to be approximately 75.4 kDa by SDS-PAGE (Fig. 1a) and the amylase activity detected corresponded to the purified protein band on native-PAGE (Fig. 1b). The molecular weights of raw starch-digesting amylases produced by microorganisms vary from 32 to 250 kDa [10]. The molecular weight of PoGA15A, which was within this molecular weight range, was similar to the RSDGs previously obtained from *C. rolfsii* (G1, G2, and G3) [18] and *Halolactibacillus* sp. [27], but significantly larger than the RSDG from *T. indicae*-*seudaticae* (42 kDa) [23].

### Scanning electron microscopy of raw cassava starch hydrolyzed by PoGA15A

Photographs of native and enzyme-hydrolyzed cassava starch granules were detected using scanning electron microscopy (SEM). Figure 2B shows that the surface of untreated cassava granules was round and smooth. When RSDG PoGA15A attacked the cassava starch granules, the surface became pitted and large, deep holes appeared on the surface (0.5 h). As the reaction time increased, larger cavities with a rugged interior surface formed at the center of the cassava starch granules (2 and 4 h) and then the granules became cracked and disrupted like an open shell sharp (8 h), which indicated that RSDG PoGA15A can efficiently degrade raw cassava starch granules. Similarly, it has also been reported that a single big hole extending into the granule interior appeared in a raw wheat starch granule hydrolyzed by an RSDG from *T. indicae*-*seudaticae* [23]. In contrast, many pinholes were observed on raw corn starch granules when an RSDG from *Cladosporium gossypiicola* was used for hydrolysis [28]. The differences in the starch granule degradation patterns by RSDGs may be attributed to variations in the raw starch granule structure and the amylose and amylopectin components in granules from different plant sources [28].

### Effect of pH and temperature on enzyme activity and stability

**Fig. 3:**
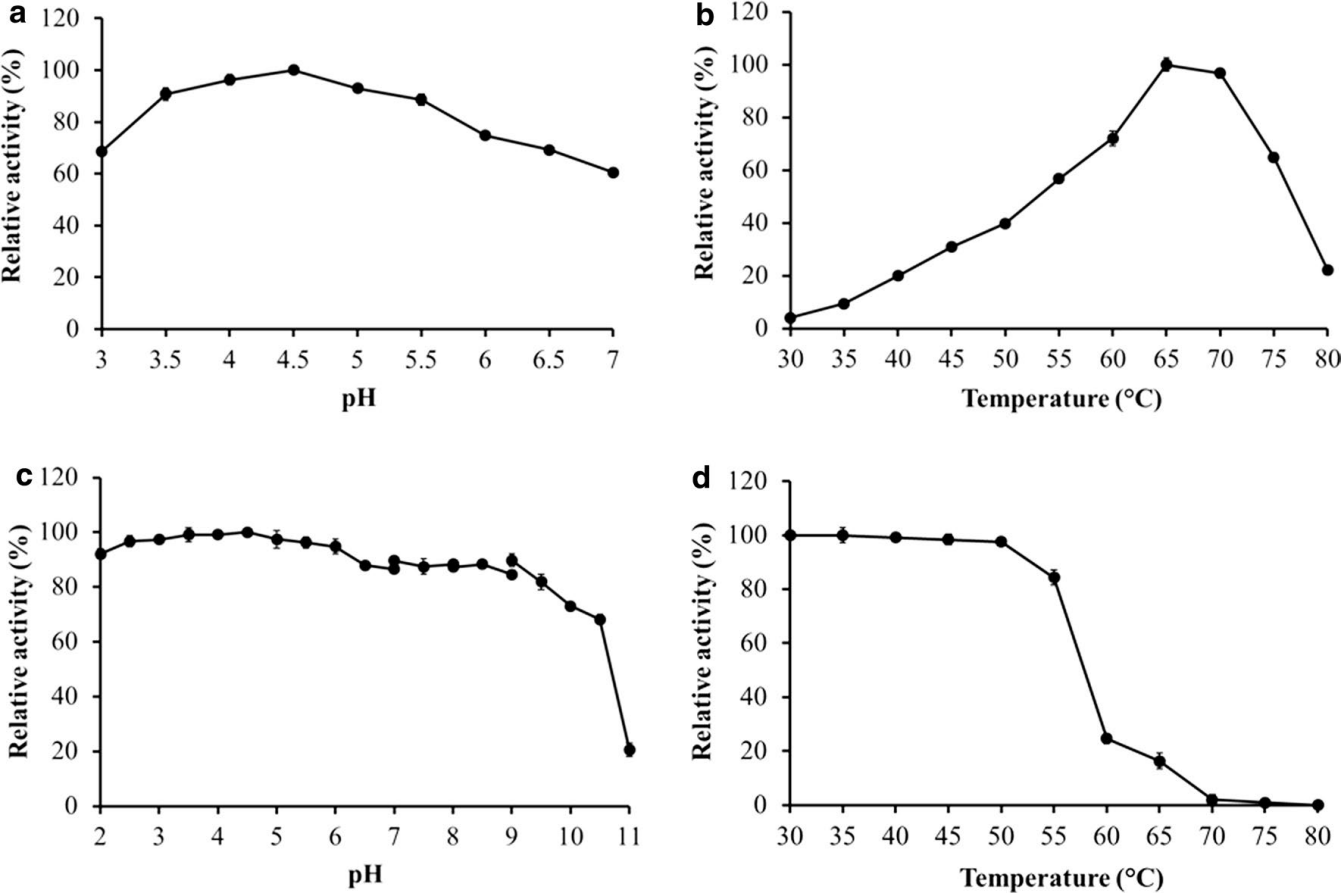
Effects of pH and temperature on enzymatic activity and the stability of the purified glucoamylase PoGA15A. Data given are mean ± standard deviation from three replicates. The results are from a representative experiment, and similar results were obtained in two other independent experiments. **a** The effect of pH on enzyme activity. The enzyme activity was assayed in a citrate–phosphate buffer (pH 3.0–7.0) at 37 °C. **b** The influence of temperature on enzyme activity. The enzyme activity was determined between 30 and 80 °C under optimum pH conditions. **c** The effect of pH on enzyme stability. The pH stability of PoGA15A was measured by pre-incubating the enzyme in various buffers for 24 h at 4 °C, and the residual enzyme activity was determined using the standard method. **d** The influence of temperature on enzyme stability. Temperature stability was determined by the standard method after pre-incubating the enzyme at pH 4.5 between 30 and 80 °C for 1 h

The effects of pH on enzyme activity were determined using a citrate–phosphate buffer with a pH ranging from 3.0 to 7.0 at 37 °C. Figure 3a shows that the purified enzyme had a maximum enzymatic activity at pH 4.5. The enzymatic activity did not decrease rapidly below or above the optimum pH. The optimum pH for purified RSDG PoGA15A was similar to previously reported RSDGs from *A. niger* (GA2) and *C. rolfsii* (G1, G2, and G3) [18, 22]. The enzyme PoGA15A showed high enzymatic activity at low acidity (pH 3.5–4.5). This matches the acid fermentation conditions for *Saccharomyces cerevisiae* [8] and improves its potential value to the starch feedstock-based ethanol fermentation industry.

The effect of temperature on enzyme activity was measured in a pH 4.5 citrate–phosphate buffer at temperatures ranging from 30 to 80 °C. The optimum temperature occurred at 65 °C and enzymatic activity significantly decreased above 70 °C (Fig. 3b). The optimum temperature for PoGA15A was similar to the RSDGs from *A. niger* [22], but lower than that of an RSDG produced by *Halolactibacillus* sp. (70 °C) [27].

To investigate the pH stability of RSDG PoGA15A, the enzyme solution was pre-incubated at 4 °C for 24 h in various pH buffers (pH 2.0–11.0) and the remaining enzymatic activity was then assayed. The enzyme was found to be highly stable between pH 2.0 and 10.5 (Fig. 3c), but enzymatic activity decreased significantly when the pH value was higher than 10.5 (Fig. 3c). The enzyme was very stable under extremely acidic conditions (pH 2.0–4.0), which indicated that the purified RSDG PoGA15A was an acidophilic enzyme. This is very rare among raw starch-digesting enzymes and is similar to an RSDG reported from *A. awamori* var. *kawachi* [17]. Moreover, such a wide range for pH stability (pH 2.0–10.5) distinguishes the PoGA15A enzyme from other raw starch-digesting amylases reported so far. For example, two RSDGs from *Acremonium* sp. endophytic fungus and the marine yeast *A. pullulans* were only stable between pH 3.0 and 7.0, and 4.0–8.0, respectively [24, 29]. The raw starch-digesting amylases from *Bacillus* sp. and *Aspergillus carbonarius* have been reported to perform well across pH ranges of 3.0–9.0 and 4.5–11.0, respectively [30, 31], but their pH stability properties were still inferior to the amylase investigated in this study.

To our knowledge, this is the first report of an RSDG with such a broad pH stability. An RSDG that possesses excellent stability over wide pH range should potentially improve enzyme production and storage. PoGA15A can perform at pH conditions that occur during starch hydrolysis with other related amylases, which extends its usage potential and reduces the need for pH adjustment. Therefore, these properties mean that RSDG PoGA15A should have a number of potential applications in the raw starch saccharification and ethanol production industries.

The thermal stability of the purified enzyme was tested by incubating it at temperatures ranging from 30 to 80 °C for 1 h. Figure 3d shows that no enzyme activity loss was observed below 50 °C and that the enzyme retained more than 80 % of its original activity at 55 °C. However, enzyme activity rapidly fell above 60 °C and no activity was detected after 1 h incubation at 75–80 °C.

### Effect of metal ions and chemical reagents on enzyme activity

Metal ions and some chemical reagents (such as detergents, chelators, and additives) are known to act as activators, inhibitors, or stabilizers. In this study, the enzymatic activity was determined at pH 4.5 and 40 °C in the presence of various metal ions or chemical reagents at a concentration of 10 mM. Additional file 1: Table S1 shows that enzyme activity was slightly stimulated by Mn^2+^ and Fe^2+^, but inhibited by Ag^+^, Cu^2+^, and SDS. Calcium ions had no obvious influence on enzyme activity. This suggested that PoGA15A was Ca^2+^-independent, which gives it an industrial application advantage. Potassium and Na^+^ ions also had little effect on enzyme activity at 10 mM. The Fe^2+^ ion has been reported to be an activator for an RSDG from *T. indicae*-*seudaticae*, but Mn^2+^ inhibited the enzyme and Ag^+^ activated it [23]. The Mn^2+^ and Cu^2+^ ions are activators for an RSDG from *Acremonium* sp., but Fe^2+^ had no effect on enzyme activity. However, EDTA could significantly inhibit enzyme activity [29]. It should be noted that the PoGA15A enzyme is Ca^2+^-independent, which is different from most of the raw starch-digesting glucoamylases [23, 24, 29]. Overall, most of the metal ions and chemical reagents did not have an obvious influence on PoGA15A activity, except SDS, Ag^+^, and Cu^2+^, which acted as inhibitors. The results also revealed that none of these ions and chemical reagents were absolutely required for catalytic activity.

### Substrate specificity and raw starch adsorbability

The substrate specificity of the purified PoGA15A from *P. oxalicum* was determined using soluble starch and a number of raw starches from various sources as substrates. The results are shown in Table 2. PoGA15A had a broad range of substrate specificities and was capable of digesting all raw starches tested. Furthermore, its enzymatic activities towards raw rice (211.3 %), corn (206.7 %), and cassava (100 %) were much higher than for other tested raw starches, including potato (90.8 %), buck wheat (59.9 %), and sweet potato (25.3 %). There is considerable economic interest in RSDGs with the capacity to digest different raw starches because they can extend the degradation ranges of raw starch sources, and directly and cost-effectively hydrolyze raw starch, which eliminates the heat pretreatment and starch liquefaction processes. An extracellular RSDG from marine yeast *A. pullulans* N13d was reported to be adsorbed on raw potato, sweet potato, and corn starch, but only raw potato starch could be actively degraded [24]. Both glucoamylase I and II from *P. oxalicum* also digested raw starches, including rice, buckwheat, corn, and potato. It had the highest enzymatic activity towards rice starch [32]. However, these two forms of glucoamylases exhibited lower activity against corn and potato starch than buckwheat starch, whereas PoGA15A displayed higher activity towards corn and potato starch than buckwheat. PoGA15A glucoamylase showed significantly higher enzymatic activity towards soluble starch (706.8 %) than the raw starches tested, which demonstrated that PoGA15A could easily hydrolyze soluble starch. Similar results have also been reported for glucoamylases from *C. rolfsii* and *P. oxalicum* [18, 32]. In contrast, a raw starch-digesting α-amylase from *Bacillus aquimaris* was able to digest raw cassava and corn starches, but had no activity against soluble starches [33].

**Table 2:** Substrate specificity of the purified PoGA15A towards soluble starch and various raw starches

Substrate	Specific activity (U/mg protein)	Relative activity (%)
Raw cassava starch	11.5 ± 0.2	100 ± 1.9
Raw rice starch	24.3 ± 0.5	211.3 ± 3.9
Raw corn starch	23.7 ± 0.4	206.7 ± 3.6
Raw potato starch	10.8 ± 0.3	90.8 ± 2.4
Raw buckwheat starch	6.9 ± 0.3	59.9 ± 2.7
Raw sweet potato starch	2.9 ± 0.2	25.3 ± 2.0
Soluble starch	81.2 ± 0.6	706.8 ± 5.2

Data are mean ± standard deviation from three replicates. The experiment was repeated three times and similar results were obtained each time

PoGA15A exhibited 41–76 % adsorbability onto the different concentrations of raw starch flours. The adsorption rate was the highest for rice and corn starch flours followed by cassava starch flour, but the adsorption rate for potato was relatively low (Table 3). The rates of adsorption increased when the concentrations of raw starch flours were elevated from 1 to 5 %. The results of the enzyme adsorbability onto different raw starch flours were consistent with the substrate specificities towards the corresponding raw starches mentioned above, which indicated that there was the correlation between the adsorption rate and raw starch hydrolysis capacity [34]. The raw starch adsorbability and raw starch hydrolysis were found to be related to the starch-binding domain, which demonstrated that this domain needs to be present in the structure of the purified PoGA15A. Gangadharan et al. [35] also reported that an α-amylase from *Bacillus amyloliquefaciens* with raw starch-digesting ability showed a strong correlation between adsorption to raw starch and hydrolysis. In contrast, a raw starch-digesting glucoamylase from *A*. *pullulans* was able to adsorb into raw potato, corn, and sweet potato starches, but only raw potato starch was degraded by the enzyme [24].

**Table 3:** Adsorbability of the purified PoGA15A towards various raw starches

Raw starch from various sources	Adsorption to raw starch granules (%)	
	1 %	5 %
Cassava	44.5 ± 1.7	56.7 ± 1.1
Corn	46.0 ± 0.4	67.3 ± 0.9
Rice	61.2 ± 1.1	76.5 ± 1.3
Potato	41.1 ± 0.7	55.4 ± 1.7

Data are mean ± standard deviation from three replicates. The experiment was repeated three times and similar results were obtained each time

### Cloning of the cDNA gene encoding the RSDG and its expression in *P. pastoris*

**Fig. 4:**
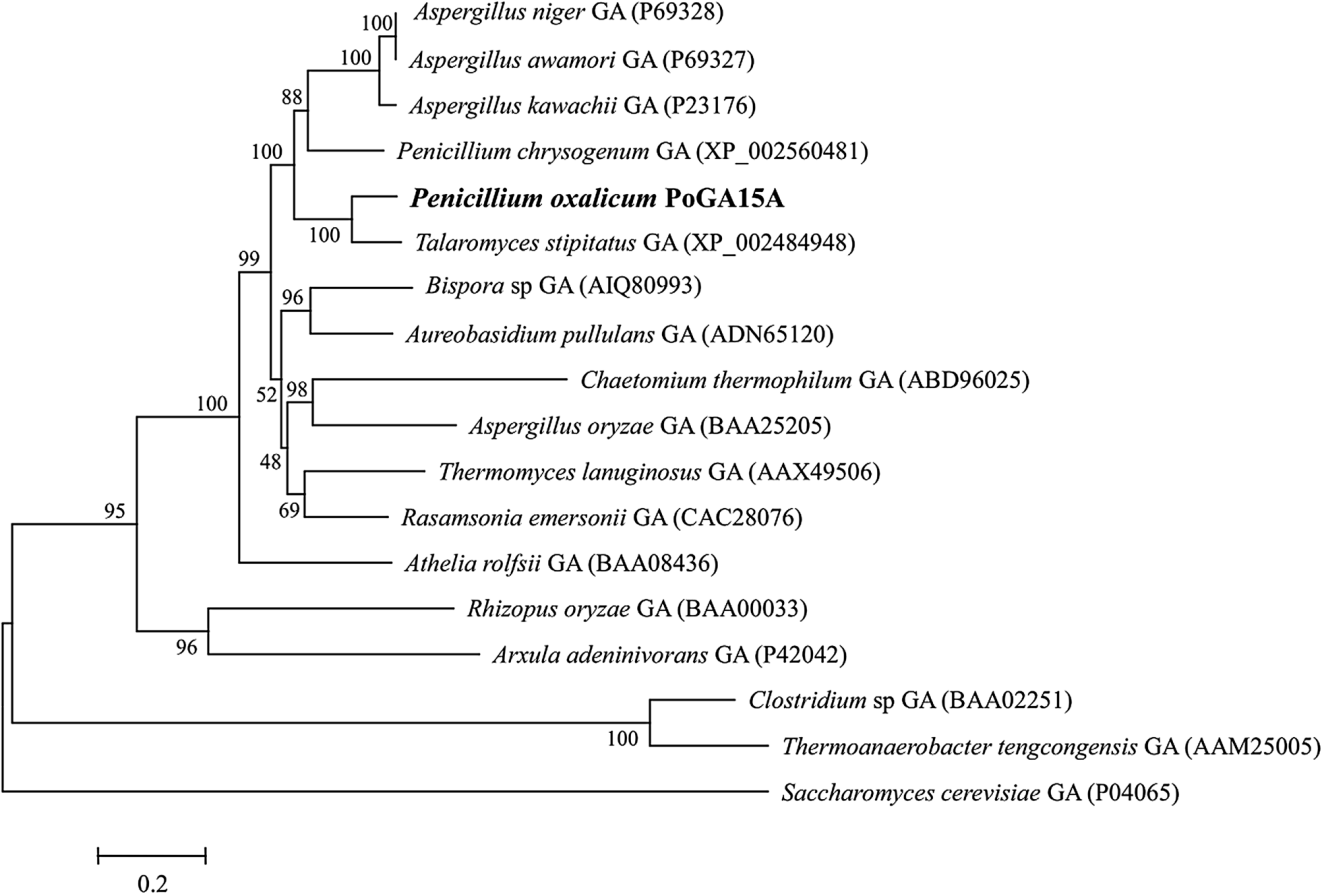
Phylogenetic analysis comparison of PoGA15A with other reported glucoamylases from bacteria and fungi. A phylogenetic tree was generated from the amino acid sequence alignments using Molecular Evolutionary Genetics Analysis (MEGA) software 4.0 and the neighbor-joining method with 1000 bootstrap replicates. All the protein sequences used for the analysis had been functionally identified and their GenBank accession numbers are shown

The purified RSDG was digested and analyzed by mass spectrometry (LTQ Orbitrap LC/MS/MS). The matched amino acid sequences of three internal peptides from *P. marneffei* ATCC 18224 (XP_002149243) and *P. oxalicum* 114-2 (EPS34453) showed 100 % sequence identity with ALANHKVYTDSFR, IGSITITSTSLAFFK, and YPEDSYYGGNPWFLSNLAAAEQLYDAIYQWNK from the glucoamylase annotated in the unigenes obtained from the *P. oxalicum* GXU20 transcriptome [26]. Therefore, an open reading frame sequence coding for the purified RSDG was obtained from the *P. oxalicum* GXU20 transcriptome data and named *PoGA15A*. The *PoGA15A* cDNA gene contained 1908 nucleotides, which coded for 635 amino acid residues. The polypeptide without the signal peptide (616 amino acid residues) showed a theoretical isoelectric point of 5.5 and had an approximate molecular weight of 65.4 kDa, which was lower than that of the purified enzyme (75.4 kDa). This indicated that glycosylation may occur during protein expression of *P. oxalicum* GXU20. BLASTP analysis revealed that the deduced amino acid sequence for the *PoGA15A* gene showed 99 % identity with a predicted glucoamylase from *P. oxalicum* 114-2 (EPS34453), 81 % identity with a putative glucoamylase from *Talaromyces marneffei* ATCC 18224 (XP_002149243), and 81 % identity with a functionally characterized glucoamylase from *Talaromyces stipitatus* ATCC 10500 (XP_002484948). Furthermore, a phylogenetic analysis of other functionally identified glucoamylases from various sources containing PoGA15A demonstrated that PoGA15A was closely related to the glucoamylase from *T. stipitatus* (XP_002484948) (Fig. 4).

The functional domain of the PoGA15A amino acid sequence was analyzed using SMART software (http://www.smart.embl-heidelberg.de), and the result showed that the sequences from 1–19, 39–454, and 533–629 belonged to the signal peptide, the catalytic domain of the glycosyl hydrolase family 15, and the starch-binding domain, respectively. A three-dimensional structure of PoGA15A was constructed using the SWISS-MODEL program based on the known crystal structure of the glucoamylase from *Hypocrea jecorina* (2vn7.1.A), which shared 56.3 % amino acid sequence identity with PoGA15A [36]. On the basis of the suggested three-dimensional structure (Additional file 2: Figure S1), the catalytic domain was predicted to mainly contain an α-helix and a β-propeller, which form the barrel structure [37]. The starch-binding domain was predicted to have a β-sandwich fold with eight β-strands distributed in the two β-sheets [38].

PoGA15A has a number of advantageous properties and a high capacity for raw starch hydrolysis. Therefore, the cDNA gene encoding the enzyme without the signal peptide was expressed in *P. pastoris* in order to obtain the enzyme preparation for raw starch hydrolysis and ethanol fermentation. The recombinant RSDG, which was named rPoGA15A, was purified by nickel-affinity chromatography and had a similar molecular weight to the native PoGA15A enzyme purified from *P. oxalicum* (Additional file 3: Figure S2), which indicated that the enzyme was correctly expressed in the heterologous expression system from *P. pastoris*. The rPoGA15A enzyme had an optimum temperature of 65 °C and an optimum pH of 4.5. The enzyme was very stable over a broad pH range (2.0–10.0), and below 50 °C. The effect of metal ions and chemical reagents, substrate specificity, and raw starch adsorption ability were also very similar to the native PoGA15A enzyme from *P. oxalicum* GXU20. HPLC analysis of raw starch hydrolysate also showed that only glucose was formed during the hydrolysis of raw starch by the rPoGA15A enzyme. The characteristics of the rPoGA15A enzyme were very similar to the native PoGA15A enzyme, which demonstrated that the rPoGA15A enzyme was correctly expressed in *P. pastoris*.

### Effective hydrolysis of raw starch flour by the recombinant rPoGA15A preparation and α-amylase

The recombinant RSDG, rPoGA15A, had a strong ability to degrade raw starches. When raw rice, corn, cassava, and potato flours at a concentration of 10 g/L were hydrolyzed by the rPoGA15A enzyme at 40 °C for 72 h, the degree of hydrolysis for each starch flour was 86.5, 71.9, 30.9, and 14.8 %, respectively (data not shown). This result showed that the hydrolysis capacity of the enzyme towards raw rice and corn starches was higher than for raw cassava and potato starches, which was consistent with the substrate specificity of the native PoGA15A enzyme towards different raw starches.

The effects of enzyme dosage on starch hydrolysis were investigated by varying the rPoGA15A enzyme dosages (0.05, 0.1, 0.2, and 0.5 U/mg raw starch) used to hydrolyze 150 g/L raw corn flour in pH 4.5 citrate–phosphate buffer at 40 °C. After 72 h hydrolysis, the hydrolysis percentages for the enzyme dosages of 0.05, 0.1, 0.2, and 0.5 U/mg raw starch flour were 65.5, 66.5, 67.0, and 70.6 %, respectively. This result showed that different enzyme dosages had no significant effect on raw starch hydrolysis, which indicated that the low enzyme dosage of 0.05 U/mg raw starch could be used for raw starch hydrolysis, and SSF of raw starch to ethanol.

Corn starch is largely produced and used as a food or industrial ingredient. It is also widely used to produce glucose–fructose syrup and bioethanol in the United States of America. Furthermore, considerable quantities of cassava starch, which is the most suitable non-grain and starch-rich feedstock, are produced in the subtropical area of China, Southeast Asian countries, Brazil, and South Africa. Therefore, raw corn and cassava starch flours were chosen for the starch hydrolysis and subsequent SSF studies. In order to more effectively hydrolyze raw starch, commercial α-amylase was added to the reaction mixture to act synergistically with the rPoGA15A during the degradation of raw starch.

**Fig. 5:**
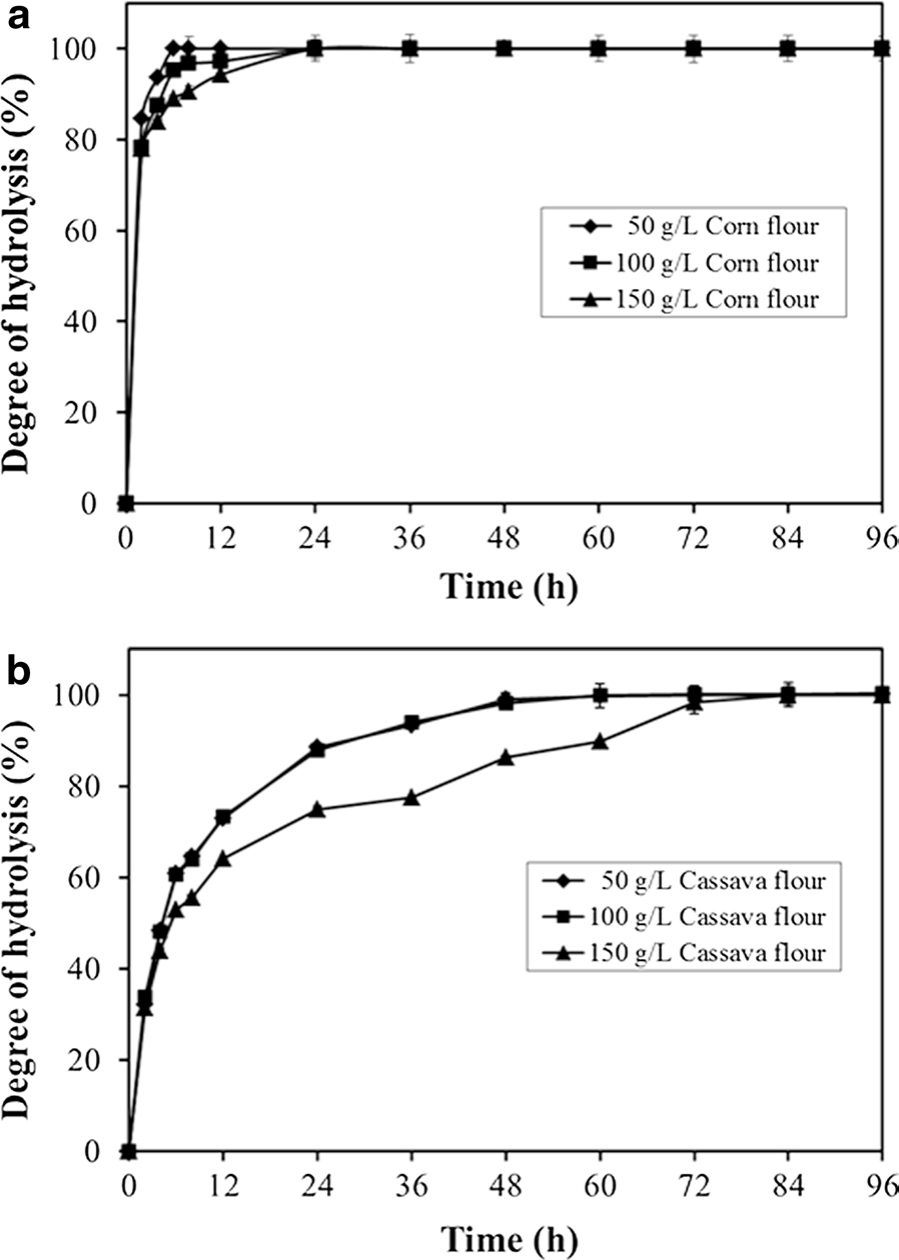
Efficient hydrolysis of raw corn flour and raw cassava flour. The experiments were conducted using the rPoGA15A from the recombinant *P. pastoris* with the addition of commercial α-amylase. Data are mean ± standard deviation from two replicates. The results shown are from a representative experiment, and similar results were obtained in two other independent experiments. The reaction took place in citrate–phosphate buffer (pH 4.5) on a shaker at 180 rpm and 40 °C. The dosage of each enzyme used was 0.05 U/mg raw flour. **a** Hydrolysis of raw corn flour at 50, 100, and 150 g/L. **b** Hydrolysis of raw cassava flour at 50, 100, and 150 g/L

The rPoGA15A could rapidly and effectively hydrolyze raw corn and cassava flours with its synergetic action of commercial α-amylase. Figure 5a shows that raw corn starch was rapidly hydrolyzed at different corn flour concentrations after using the rPoGA15A preparation in combination with commercial α-amylase. The amount of starch hydrolyzed at corn flour concentrations of 50, 100, and 150 g/L could reach up to 84.6, 78.3, and 77.9 %, respectively, after a very short incubation time of 2 h. The starches in corn flour of 50 g/L were 100 % hydrolyzed after 6 h. After 12 h, the starches in corn flour of 100 and 150 g/L were almost completely hydrolyzed, and the percentage of starch hydrolyzed at the two concentrations increased to 100 % at 24 h (Fig. 5a). These results demonstrated that raw corn starch was efficiently hydrolyzed by the synergetic action of the rPoGA15A enzyme and commercial α-amylase.

The rPoGA15A enzyme or commercial α-amylase alone could not sufficiently and quickly hydrolyze raw cassava starch at the different concentrations of raw cassava flour, and reached no more than 37 % hydrolysis after 96 h degradation (data not shown). When both the rPoGA15A enzyme and α-amylase were used to hydrolyze the raw cassava starch, the hydrolysis efficiency significantly improved. Figure 5b shows that the amounts of starch hydrolyzed at cassava flour concentrations of 50, 100, and 150 g/L were 84.4, 87.8, and 74.9 %, respectively, at 24 h. The raw starches in cassava flour at concentrations of 50 and 100 g/L had been almost entirely hydrolyzed at 48 h, and reached 100 % hydrolysis after 60 h. The raw starches in cassava flour at concentration of 150 g/L were almost completely degraded after 72 h and reached 100 % hydrolysis at 84 h.

The synergistic use of the rPoGA15A and commercial α-amylase remarkably improved raw starch hydrolysis. Similarly, an RSDG from *Penicillium* sp. X–1 significantly hydrolyzed 15 % (w/v) of raw corn starch slurry after it was supplemented with commercial α-amylase at 65 °C and pH 6.5, and achieved much higher hydrolysis levels (92.4 %) after 2 h compared to using the enzyme alone [19]. Matsubara et al. [39] investigated the raw starch hydrolysis mechanisms used by α-amylase (Amyl III) and glucoamylase (GA I) from *Aspergillus awamori* KT-11, and thought that GA I initially degraded the starch granule surface to form many holes and released glucose as a product. Then Amyl III could adsorb to the holes to further digest the starch granule producing large oligosaccharides as the main hydrolysis products [39]. Raw corn starch was more susceptible to hydrolysis than raw cassava starch (Fig. 5). Raw starches from cereals are more rapidly and completely hydrolyzed by raw starch-digesting enzymes than those from roots or tubers [12]. This result might be attributed to differences in structures and/or surface area effects between corn and cassava starch granules [40].

To our knowledge, the hydrolysis performance of rPoGA15A with α-amylase at the relatively low temperature of 40 °C was significantly better in terms of hydrolysis speed and hydrolysis percentage than the activities reported by other studies using raw starch-digesting enzymes. A 95 % hydrolysis of 150 g/L raw cassava starch slurry was realized by the crude enzyme from *P. oxalicum* GXU20 after 72 h incubation at 40 °C [20]. When a commercial raw starch-digesting enzyme was used, raw corn and cassava starches in starch slurry at concentration of 25 % (w/v) were degraded by the STARGEN™ 001 enzyme produced by Genencor International (Palo Alto, CA, USA) at 35 °C, but only 52.6 and 35.4 % hydrolysis of the two starches was achieved after 24 h, respectively [41]. Li et al. [42] also reported that the hydrolysis of raw corn starch by STARGEN™ 002 from the Genencor reached 72 % at 30 °C after 96 h when 30 % of the starch slurry was pre-heated to the sub-gelatinization temperature (61 °C) for 30 min in the presence of urea. Raw cassava starch at 100 g/L was hydrolyzed by raw starch-digesting α-amylase from *Laceyella sacchari* LP175 bacteria at 50 °C after 12 h and reached 36.8 % hydrolysis. The hydrolysis was synergistically improved and reached up to 70 % after the addition of commercial glucoamylase [43]. An extracellular enzyme from *Microbacterium aurum* could digest 5 % raw cassava starch at 37 °C and 76 % hydrolysis was obtained after a long incubation time of 148 h [40]. Therefore, the good raw starch hydrolysis performance of PoGA15A gives it many potential applications in the starch hydrolysis, food processing, and ethanol production industries.

### Efficient simultaneous saccharification and fermentation (SSF) of raw starch flour to ethanol

**Fig. 6:**
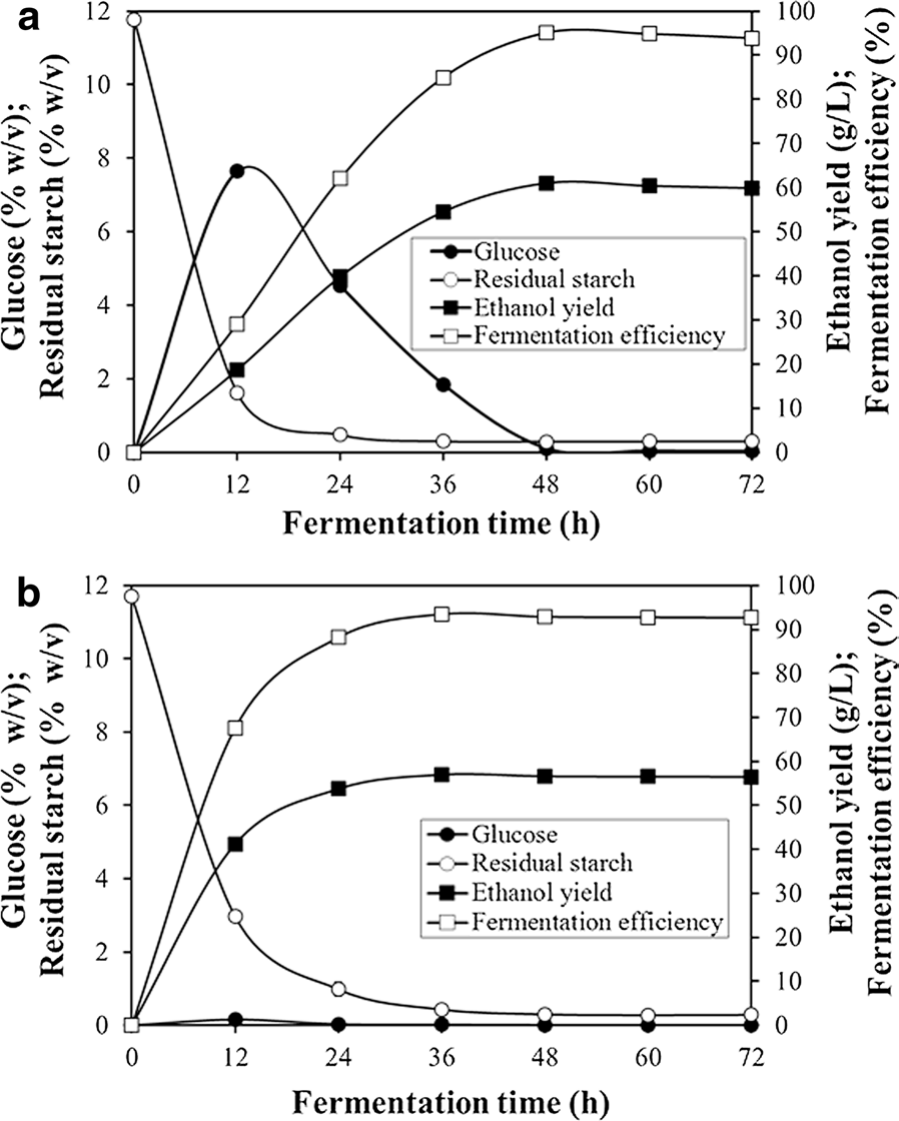
Simultaneous saccharification and fermentation of raw corn flour and raw cassava flour to ethanol. The experiments were carried out using the rPoGA15A from recombinant *P. pastoris* and commercial α-amylase at a raw flour concentration of 150 g/L. The dosage of each enzyme used was 0.05 U/mg raw flour. The fermentation was conducted at 40 °C. Data are mean ± standard deviation from two replicates. The results shown are from a representative experiment, and similar results were obtained in two other independent experiments. **a** Simultaneous saccharification and fermentation of raw corn flour to ethanol. **b** Simultaneous saccharification and fermentation of raw cassava flour to ethanol

Raw corn flour and raw cassava flour were used as fermentation substrates to investigate the SSF of raw starch to ethanol by rPoGA15A and commercial α-amylase. The SSF was conducted at 40 °C, and the concentrations of produced glucose, residual starch, and ethanol were measured at 12 h intervals. Figure 6a shows that raw corn starch was rapidly hydrolyzed after 12 h, and the amount of glucose produced in the mixture increased significantly. Simultaneously, ethanol was also rapidly produced by the yeast. As the fermentation time increased, the glucose in the mixture gradually fell, and the ethanol yield quickly increased. The maximum ethanol yield (61.0 g/L) was achieved in just 48 h. A high fermentation efficiency of 95.1 % was obtained, and the residual starch (less than 0.2 %, w/v) and glucose concentration (less than 0.1 %, w/v) were extremely low in the mixture at 48 h, which indicated that the SSF of raw corn flour to ethanol was very rapid and efficient.

The SSF of raw cassava flour to ethanol also occurred rapidly and successfully. Figure 6b shows that raw cassava starch in the slurry was quickly degraded by the enzyme after 12 h. However, little glucose was released into the reaction mixture (about 0.15 %, w/v). Consequently, a large amount of ethanol was produced by the yeast. This result demonstrated that glucose from raw cassava starch saccharification was rapidly utilized by the yeast to produce ethanol, and the low levels of glucose in the mixture could further enhance raw starch hydrolysis, which suggested that raw starch saccharification and ethanol fermentation occurred simultaneously and matched very well. At 36 h, the maximum ethanol yield and fermentation efficiency reached 57.0 g/L and 93.5 %, respectively. The residual starch (less than 0.3 %, w/v) and glucose concentration (less than 0.02 %, w/v) were also extremely low. There was no increase in the yield of ethanol after 36 h, which showed the excellent SSF performance when converting raw cassava flour to ethanol.

The SSF of raw cassava flour to ethanol was accomplished after 36 h, which was earlier than that of raw corn flour (48 h). The reason for this result might be the mutual coordination between the enzyme saccharification of starch and ethanol fermentation. Raw corn starch was hydrolyzed to glucose more quickly than the rate of yeast fermentation, and thus a large amount of glucose remained in the mixture, especially at 12 h. This might lead to inhibition of the following ethanol fermentation step due to the high glucose concentration, which may be eliminated by reducing the amount of enzyme used. However, enzyme saccharification of raw cassava starch matched ethanol fermentation, so very low amounts of glucose were present during the fermentation process and a more rapid fermentation speed was achieved compared to corn starch.

People have shown great interest in the direct saccharification and fermentation of raw starch to ethanol without cooking, due to the significant reduction in energy consumption and production costs [8]. Two main representative technologies are SSF and the consolidated bioprocess (CBP) for the ethanol fermentation of raw starch [7, 8]. Table 4 compares the fermentation of raw corn and cassava starches to ethanol under conditions that were similar to those reported in previous studies. The fermentation efficiency of raw corn starch reported in this study was much higher than the efficiencies achieved by previous studies. Han et al. [44] used a crude enzyme from *A. niger* and a yeast for the SSF of raw corn starch, and obtained a high fermentation efficiency of 95.9 %. The fermentation efficiency was very similar to this study (95.1 %), but a longer fermentation time (72 h) was needed for the SSF of raw corn starch to ethanol than our study (48 h). The fermentation efficiency of raw cassava starch in this study was higher than in previous studies, and the fermentation time was also shorter (Table 4). Therefore, the very effective SSF of raw corn and cassava starches to ethanol without cooking suggests that rPoGA15A has considerable potential in the ethanol production industries.

**Table 4:** Fermentation of raw corn and cassava starches to ethanol under different conditions

Enzymes/fermentation microorganisms	Raw starch sources	Fermentation conditions, mode, and finish time	Fermentation efficiency (%)	References
rPoGA15A and α-amylase/*S. cerevisiae*	Corn	40 °C, pH 4.0, SSF^a^, 48 h	95.1	This study
rPoGA15A and α-amylase/*S. cerevisiae*	Cassava	40 °C, pH 4.0, SSF, 36 h	93.5	This study
*Rhizopus* sp. extract/*S. cerevisiae*	Corn	30 °C, SSF, 48 h	94.5	[45]
*Aspergillus* sp. extract/*S. cerevisiae*	Corn	30 °C, pH 3.5, SSF, 96 h	92.7–94.0	[46]
*Chalara paradoxa* extract/*S. cerevisiae*	Corn	30 °C, pH 5.0, SSF, 120 h	63.5–86.8	[47]
*Chalara paradoxa* extract/*S. sake*	Corn	30 °C, pH 5.0, SSF, 120 h	81.1–92.1	[47]
*A. niger* amylases/yeast	Corn	30 °C, pH 4.1–4.3, SSF, 72 h	95.9	[44]
STARGEN 001/*S. cerevisiae*	Corn	30 °C, pH 4.0, SSF, 72 h	88.4	[9]
STARGEN 001/*S. cerevisiae*	Corn	32 °C, pH 3.7, SSF, 70 h	91.3	[48]
STARGEN 002/*S. cerevisiae*	Corn	32 °C, pH 3.7, SSF, 70 h	85.9	[48]
STARGEN 001*/S. cerevisiae*	Corn	35 °C, pH 5.0, SSF, 72 h	83.4	[49]
*S. cerevisiae* displaying amylolytic enzymes	Corn	30 °C, CBP^b^, 20 batches (one batch for 24 h)	76.6	[50]
Engineering *S. cerevisiae*	Corn	30 °C, CBP, 120-240 h	61–80	[51]
*P. oxalicum* extract/*S. cerevisiae*	Cassava	40 °C, pH 4.0, SSF, 48 h	92	[20]
*A. kawachii* extract/yeast	Cassava	37 °C, pH 4.0, SSF, 85 h	92.3	[52]
*Rhizopus* koji	Cassava	35 °C, pH 4.5, SSF, 96 h	72.3–83.5	[53]
*Rhizopus* koji	Cassava	35 °C, pH 4.5–5.0, SSF, 288 h	74.5–85.5	[54]
Engineering *K. marxianus*	Cassava	42 °C, pH 4.8–5.6, CBP, 96 h	78.3	[55]

^a^ *SSF* represents simultaneous saccharification and fermentation

^b^CBP represents consolidated bioprocessing

### Effect of the starch-binding domain on the enzymatic activity of rPoGA15A

In order to investigate what is responsible for the strong raw starch-degrading capacity of rPoGA15A, a partial *PoGA15A* gene that did not contain the SBD coding sequence was over-expressed in *P. pastoris*. The recombinant protein was purified using a nickel-affinity column and analyzed on SDS-PAGE. Additional file 4: Figure S3 shows that the molecular weight of the protein was 62.4 kDa, which is slightly larger than the theoretical prediction. This indicated that protein glycosylation may occur during the heterologous expression process.

The specific activities of the purified enzymes towards different starches were determined at a starch concentration of 1 % in citrate–phosphate buffer (pH 4.5) at 40 °C. Table 5 shows that the substrate specificity of the enzyme lacking SBD significantly decreased for all the starches tested compared to the wild-type enzyme rPoGA15A. The specific activity of the mutant enzyme towards raw rice (18.2 %) and corn starch (30.3 %) decreased more than for raw cassava (47.4 %) and potato starch (51.2 %). The specific activity towards soluble starch (71.9 %) showed a smaller reduction in enzymatic activity than for the raw starches. No adsorption ability towards raw starches was observed for the mutant enzyme lacking the SBD domain (data not shown). Hence, the results for the substrate specificity and raw starch adsorbability tests demonstrated that the SBD in rPoGA15A plays a very important role during starch hydrolysis, especially in the efficient degradation of raw starch.

**Table 5:** Substrate specificity of an rPoGA15A mutant without SBD towards different starches when compared to the wild-type enzyme rPoGA15A

Substrate	Specific activity for wild-type enzyme rPoGA15A (U/mg protein)	Specific activity for mutant enzyme rPoGA15A lacking SBD (U/mg protein)	Relative activity (%)^a^
Raw cassava starch	11.2 ± 0.9	5.3 ± 0.2	47.4 ± 1.7
Raw rice starch	24.7 ± 0.4	4.5 ± 0.1	18.2 ± 0.5
Raw corn starch	22.7 ± 0.7	6.8 ± 0.1	30.3 ± 0.3
Raw potato starch	9.9 ± 0.5	5.1 ± 0.2	51.2 ± 1.4
Soluble starch	80.5 ± 0.7	57.9 ± 0.4	71.9 ± 0.5

^a^Values were shown as percentages, which represent the substrate specific activity of the mutant enzyme rPoGA15A without SBD × 100/the corresponding substrate specific activity of the wild-type enzyme rPoGA15A

Only about 10 % of the amylolytic enzymes possessing a distinct SBD are capable of raw starch degradation [56]. Currently, SBDs can be divided into nine carbohydrate-binding module (CBM) families based on amino acid sequence similarities [38]. The SBD of the enzyme is able to bind raw starch granules, which can increase local substrate concentration at the active site of the enzyme catalytic center [57]. Some enzyme SBDs may also disrupt the surface of the starch structure, thereby enhancing the efficiency of raw starch hydrolysis [58].

An α-amylase (AMY-CS2) from yeast *Cryptococcus* sp. S-2 also has raw starch-digesting activity. Nevertheless, the mutant AMY-CS2 α-amylase lacking an SBD lost not only its capacity for raw starch degradation and adsorption, but also its thermal stability [59]. Conversely, the SBD from *A. niger* was added to a glucoamylase from *S. cerevisiae* without an SBD, and thus conferred the hybrid enzyme capabilities for substrate binding and insoluble starch hydrolysis [60]. Therefore, the SBD plays a very important role in raw starch hydrolysis, and protein engineering of the SBD or replacement of the SBD in an original enzyme with strongly adsorbing SBDs from other enzymes may provide another alternative way to further enhance the activity of PoGA15A raw starch degradation.

## Conclusions

A novel raw starch-digesting glucoamylase PoGA15A displaying a high capacity for raw starch degradation was purified from *P. oxalicum* GXU20 and biochemically characterized. Its cDNA was cloned and heterologously expressed in *P. pastoris*. The PoGA15A enzyme was most active at pH 4.5 and 65 °C. It showed remarkable stability over a wide pH range (2.0–10.5), and the enzymatic activity was not adversely influenced by most of the metal ions and chemical reagents tested. The enzyme showed broad substrate specificity against raw starches and could quickly and efficiently hydrolyze raw corn and cassava flours at different concentrations with the addition of α-amylase. The SSF of raw corn and cassava flours to ethanol was rapidly and efficiently accomplished by the rPoGA15A enzyme with the addition of α-amylase. Analysis of a mutant rPoGA15A enzyme that lacked an SBD revealed that the SBD was mainly responsible for the high raw starch degradation capacity of the rPoGA15A enzyme. This study has improved understanding of a novel RSDG, and its excellent properties mean that the enzyme has great potential in the starch hydrolysis and ethanol production industries.

## Methods

### Chemicals and materials

Hiprep 16/10 phenyl and Source 15S 4.6/100 PE were purchased from GE Healthcare Life Sciences (Uppsala, Sweden). Raw starches (cassava, rice, corn, potato, sweet potato, and buckwheat) were purchased from a local market in Nanning, China. The raw starch substrates used for enzymatic activity assay were washed four times with ultra-pure water. The starch contents of raw flours were determined following a method previously described by Lin et al. [20]. The starch contents of the raw flours from cassava, rice, corn, and potato used for hydrolysis and simultaneous saccharification and fermentation of raw starch to ethanol were determined to be 78.0, 75.5, 78.4, and 76.6 %, respectively. Soluble starch was purchased from Sigma-Aldrich (St. Louis, MO, USA). The α-amylase (Suhong^®^ AA XP) was obtained from Novozymes (Tianjin, China). All the chemicals used were of analytical grade and were obtained from commercially available sources.

### Microbial strains and culture conditions

*Penicillium* sp. GXU20 was a fungal strain previously isolated from forest soil taken from Shiwandashan Mountain in Guangxi Zhuang Autonomous Region of China [20]. It was further identified as *Penicillium oxalicum* based on the β-tubulin gene, the ITS sequence, and its morphological characteristics [26]. The GXU20 strain was conserved in the China General Microbiological Culture Collection Center (CGMCC) with preservation number CGMCC No. 3690. This strain was grown on potato dextrose agar (PDA) medium at 28 °C for 6 days. A spore solution was then obtained by washing the mycelium with sterilized water and this was used as the inoculum. A spore suspension of 1 mL (10^8^ spores/mL) was added to 150 mL of culture medium in a 500 mL Erlenmeyer flask. The culture medium used for enzyme production was composed of (w/v): 3.5 % wheat bran, 2.5 % soybean meal, 0.3 % KH_2_PO_4_, 0.02 % MgSO_4_·7H_2_O, 0.00255 % FeSO_4_·7H_2_O, and 0.013 % CaCl_2_. The medium was adjusted to pH 5.5. The cultures were maintained at 28 °C on a rotary shaker at 180 rpm. After 6 days of incubation, the culture medium was centrifuged at 4 °C and 4000×*g* for 20 min and the supernatant was then used for enzyme purification.

*Escherichia coli* DH5α and *P. pastoris* GS115 strains were used for gene cloning and heterologous gene expression, respectively. A thermo-resistant dried yeast (*Saccharomyces cerevisiae*) purchased from the Angel Yeast Co., Ltd. (Yichang, China) was used for the simultaneous saccharification and fermentation of raw starch to ethanol.

### Purification of the raw starch-digesting enzyme

The culture supernatant was obtained by centrifuging as described above. Pre-chilled ethanol was added to the supernatant over ice until it reached 33 % saturation. After the removal of precipitation by centrifugation (10,000×*g*, 20 min), more ethanol was added to bring the supernatant to 67 % saturation. The solution was stored overnight at 4 °C and then centrifuged. The precipitate was dissolved in 50 mM phosphate buffer containing 1.5 M ammonium sulfate (pH 7.0) and then filtered through a 0.45 µm membrane filter.

The purification of the raw starch-digesting enzyme from *P. oxalicum* GXU20 was performed at room temperature using a fast protein liquid chromatography system (AKTA Purifier Explorer, Pharmacia, Uppsala, Sweden). The sample was loaded onto a Hiprep 16/10 phenyl column (GE Healthcare, Uppsala, Sweden) equilibrated with 50 mM phosphate buffer containing 1.5 M (NH_4_)_2_SO_4_ at pH 7.0. The column was washed with equilibration buffer until no absorbance at 280 nm was observed in the eluent. The proteins were then eluted using (NH_4_)_2_SO_4_ (400 mL) across a linear concentration gradient of 1.5–0 M in the same buffer at a flow rate of 4 mL/min. Then, 5 mL fractions were collected and assayed for enzyme activity.

Fractions with raw starch-digesting enzyme activity were pooled, concentrated by ultrafiltration in an Amicon Ultra-15 (MWCO 10,000, Amicon, EMD Millipore, Billerica, MA, USA), and dialyzed against 25 mM sodium acetate buffer (pH 4.0). The sample was then loaded onto a source 15S 4.6/100 PE column (GE Healthcare, Uppsala, Sweden) pre-equilibrated with 25 mM sodium acetate buffer at pH 4.0. The adsorbed enzyme was eluted using a linear NaCl concentration gradient (0–1 M) in the same buffer with a 1 mL/min flow rate. Protein purity was analyzed by sodium dodecyl sulfate-polyacrylamide gel electrophoresis (SDS-PAGE). The purified active fractions were collected and used for further analysis.

### Enzyme activity assay

The raw starch-digesting enzyme activity was determined by measuring the reducing sugars released during raw starch hydrolysis. The reaction mixture, containing 450 μL of 1 % raw cassava flour in 0.1 M citrate–phosphate buffer (pH 4.5) and 50 μL enzyme solution, was incubated at 40 °C for 30 min and the reaction was stopped by heating for 10 min in boiling water. A control sample was incubated for 10 min in boiling water to inactivate the enzyme and incubated under the same conditions as above. The reducing sugar content was measured using the dinitrosalicylic (DNS) acid method [61]. One unit of enzymatic activity was defined as the amount of enzyme that produced 1 μmol of reducing sugar (equivalent to 1 μmol glucose) per min under standard assay conditions mentioned above.

### HPLC analysis of raw cassava starch hydrolysates by the purified enzyme and scanning electron microscopy (SEM) observation of raw cassava starch granules treated with the purified enzyme

The reaction mixture, containing 0.3 mL of purified enzyme solution (6 U) and 1.5 mL of 2 % raw cassava flour resuspended in pH 4.5 citrate–phosphate buffer, was incubated at 40 °C. An aliquot of the reaction mixture (300 μL) was removed at time intervals of 0.5, 2, 4, and 8 h. The supernatant used for hydrolysis product analysis was obtained by centrifugation at 12,000×*g* for 5 min and was then incubated for 10 min in boiling water. The precipitate containing the raw cassava granules was washed with pure ethanol three times followed by drying at 35 °C to a constant weight. It was then used for the SEM study [20].

The reaction products were analyzed by high-performance liquid chromatography (HPLC) on an instrument equipped with a refractive index detector as described previously (LC-10AT, Shimadzu, Tokyo, Japan) [26]. A carbohydrate column (7.8 × 300 mm; Benson Polymeric Inc., NV, USA) was maintained at 80 °C and ultra-pure water was used as the mobile phase at a flow rate of 1 mL/min. For the SEM study, raw cassava starch granules treated with the purified enzyme at different time intervals were attached to the SEM holder and then coated with gold. The micrographs of the samples were obtained using a SU8020 scanning electron microscope (Hitachi, Ltd., Tokyo, Japan).

### Protein concentration determination and gel electrophoresis

Protein concentration was determined by the bicinchoninic acid (BCA) method using the Pierce Protein Assay Kit from Thermo Fisher Scientific (Waltham, MA, USA) [62]. Bovine serum albumin was used as a standard.

SDS-PAGE (sodium dodecyl sulfate-polyacrylamide gel electrophoresis) analysis was performed using 12 % polyacrylamide to determine protein purity and the molecular mass of the purified enzyme as described previously [63]. Proteins gels were stained with Coomassie Brilliant Blue R-250 and destained with 10 % (w/v) acetate solution. An unstained protein marker (Thermo Fisher Scientific) was used as a molecular mass standard. Native-PAGE containing 10 % polyacrylamide and 1 % soluble starch was used for the enzyme activity staining analysis. The gel was stained with KI/I_2_ reagent as described previously [64].

### Effects of pH and temperature on enzyme activity

The effect of pH on enzyme activity was measured at 37 °C in 0.1 M citrate–phosphate buffer at pH values ranging from 3.0 to 7.0. The effect of temperature on enzyme activity was investigated at temperatures ranging from 30 to 80 °C in citrate–phosphate buffer (pH 4.5). The pH stability was determined by pre-incubating the purified enzyme at various pHs (2.0–11.0) at 4 °C for 24 h and the residual enzyme activity was measured under standard conditions as described above. The buffers (0.1 M) used were citrate–phosphate buffer (pH 2.0–7.0), phosphate buffer (pH 7.0–8.0), Tris–HCl (pH 8.0–9.0), and glycine–NaOH (pH 9.0–11). Thermal stability was determined by pre-incubating the purified enzyme in the citrate–phosphate buffer (pH 4.5) at different temperatures (30–80 °C) for 1 h, and the residual enzyme activity was determined under standard assay conditions.

### Effects of different metal ions and chemical reagents on enzyme activity

The influence of various metal ions and chemicals on the enzyme activity was determined by measuring enzyme activity under standard conditions in the presence of various metal ions and chemical reagents (K^+^, Na^+^, Mg^2+^, Ca^2+^, Ni^2+^, Zn^2+^, Li^+^, Mn^2+^, Cu^2+^, Fe^2+^, Fe^3+^, Co^2+^, Ag^+^, EDTA, SDS, Tween 80, and Triton X-100) at a final concentration of 10 mM. Enzyme activity assayed in the absence of any additives was taken as 100 %.

### Substrate specificity and raw starch adsorbability

The substrate specificity of the purified enzyme was measured using various kinds of starch (cassava, rice, corn, potato, buckwheat, sweet potato, and soluble starch) as substrates. Enzyme activity against different substrates was performed in a reaction mixture containing 1 % of each starch flour in citrate–phosphate buffer (pH 4.5) at 40 °C for 30 min. The enzyme activity towards raw cassava starch was taken as 100 %.

To determine the adsorption of the enzyme onto raw starch, 50 μL of the purified enzyme solution was mixed with 450 μL of citrate–phosphate (pH 4.5) containing 1 or 5 % of each raw starch flour. The mixture was incubated at 40 °C for 30 min with occasional shaking. After centrifugation at 8000×*g* for 5 min, the enzyme activity of the supernatant was determined as described above. The percentage adsorption was calculated according to the following equation: Adsorption (%) = (A−B) × 100/A, where A is the enzyme activity of the control without raw starch and B is the residual enzyme activity in the supernatant after adsorption.

### Cloning of the cDNA encoding the purified enzyme and its expression in *Pichia pastoris*

The purified enzyme bands were excised from the SDS-PAGE gels. The proteolytic peptides were analyzed using a nanoAcquity UPLC system coupled with a LTQ Orbitrap XL ETD mass spectrometer (Thermo Fisher Scientific) at the Institute of Biomedical Sciences of Fudan University (Shanghai, China). The transcriptome sequencing data for *P*. *oxalicum* GXU20 under the culture conditions mentioned above had been obtained from a previous study [26].

The complete open reading frame encoding the enzyme was identified in the transcriptome unigenes of *P*. *oxalicum* GXU20 by combining the results of the purified enzyme’s mass spectrometric analysis with the obtained transcriptome sequencing data for *P*. *oxalicum* GXU20 [26, 65].

Total RNA was extracted from the mycelia of *P*. *oxalicum* GXU20, and cDNA was synthesized using a PrimeScript™ 1st Strand cDNA Synthesis Kit (Takara, Dalian, China), following the manufacturer’s instructions. Based on the full-length sequence obtained from the transcriptome data, the cDNA gene was amplified by PCR using cDNA as the template and the forward primer (5′-ATGTCTCGACTTCTCTACGCAC-3′) and reverse primer (5′-TTAGCGCCAGGTGTCGTTCT-3′). PCR conditions were as follows: 95 °C, 5 min, followed by 30 cycles of 60 s at 95 °C, 30 s at 54 °C, 60 s at 72 °C, and finally, a 10 min extension at 72 °C. The PCR products were sequenced and the amplified sequences showed 100 % identity with the sequences obtained from the transcriptome data. The cDNA coding the PoGA15A enzyme was analyzed and the signal peptide sequence of the enzyme was identified using SignalP 4.1 software (http://www.cbs.dtu.dk/services/SignalP/) [66]. In order to efficiently express the *PoGA15A* gene in *P. pastoris*, the *PoGA15A* gene without the sequence coding for the signal peptide underwent codon optimization by Generay Biotech Co., Ltd. (Shanghai, China). The optimized sequence with the addition of *Avr*II and *Not*I restriction sites, and the sequence coding 6× His-tag was also synthesized. The synthesized fragment was double digested with *Avr*II and *Not*I (Takara) and ligated into the *Avr*II and *Not*I sites of the pPIC9k vector (Invitrogen, Carlsbad, CA, USA), where it was fused in-frame with the α-factor signal peptide from *P. pastoris*. The resulting plasmid, pPIC9k-PoGA15A, was transformed into *E. coli* DH5α competent cells, and sequencing was performed to verify the construct by Shenzhen Huada Gene Technology Co., Ltd. (Shenzhen, China). The pPIC9k-PoGA15A vector was linearized with *Sac*I, and transformed into *P. pastoris* GS115 (Invitrogen) by electroporation using a Gene Pulser Xcell (Bio-Rad, Hercules, CA, USA) following the manufacturer’s instructions. Transformants were screened based on their ability to grow on minimal dextrose (MD) medium plates, and then inoculated onto yeast extract peptone dextrose (YPD) medium plates. The cultures were grown in the presence of increasing concentrations of Geneticin G418 (1.0, 1.5, 2.0, and 2.5 mg/mL) to screen for multicopy gene recombinants. They were then incubated for 3 days, and integration of the *PoGA15A* gene into the *P. pastoris* GS115 genome was confirmed by PCR using 5′ AOX1 and 3′ AOX1 primers [67]. Yeast transformants were initially grown in 5 mL of yeast extract peptone glycerol (YPG) medium on an orbital shaker at 250 rpm and 28 °C until the OD_600_ reached 10. Cell pellets were harvested by centrifugation at 1000×*g* for 5 min, resuspended in 40 mL of BMMY medium with 1 % methanol (v/v), and then incubated at 28 °C with shaking at 250 rpm. Methanol was added to make a final concentration of 1 % every 12 h to maintain the induction. The *P. pastoris* GS115 strain transformed with the empty vector was used as a control for background expression analysis. Enzymatic activity from the culture supernatant was detected using raw cassava as the substrate and following the method described above. The recombinant protein in the extracellular supernatant of the expression culture media was first purified by affinity chromatography with nickel–nitrilotriacetic acid agarose resin (Ni–NTA, Qiagen). Then the secreted and purified proteins were analyzed by SDS-PAGE. The purified recombinant glucoamylase with high raw starch degradation abilities was also biochemically characterized according to the methods previously mentioned above.

### Hydrolysis of raw starch flour by the recombinant rPoGA15A preparation and α-amylase

Different concentrations of the raw corn or cassava flour suspensions (50, 100, and 150 g/L) were prepared in citrate–phosphate buffer (pH 4.5), and the rPoGA15A enzyme solution, concentrated from the *P. pastoris* expression supernatant, and commercial α-amylase were added to 0.05 U per mg of each raw starch flour. The reaction mixture, with a total volume of 1.0 mL, was incubated at 40 °C with shaking at 150 rpm. After incubation of the mixtures, samples were withdrawn at various time intervals and centrifuged for 5 min at 12,000×*g*. The reducing sugar content of the supernatant was determined by the DNS method using glucose as the standard. The percentage in hydrolysis of raw starch was defined by the following formula: *R*_h_ (%) = (*A*_1_/*A*_0_) × 0.9 × 100, where *A*_1_ was the amount of reducing sugar in the supernatant after enzymatic hydrolysis and *A*_0_ was the amount of raw starch before the reaction [68].

### Simultaneous saccharification and fermentation (SSF) of raw starch flour to ethanol

SSF experiments were conducted using raw corn and cassava flours without the high temperature cooking and enzyme liquefaction pretreatments. In order to prevent microbial contamination in SSF, the raw corn and cassava flours were sterilized by Co^60^ radiation at 20 kGy. The fermentation system comprised 150 g/L of raw starch flour, rPoGA15A (0.05 U/mg raw starch flour), commercial α-amylase (0.05 U/mg raw starch flour), 3 g/L urea, and 1 g/L yeast. The mixture (20 mL) was adjusted to pH 4.0 by 2 M HCl and incubated in 50 mL sterilized conical flasks at 40 °C without agitation. Then, 1.5 mL aliquots of the slurry were withdrawn at 12 h time intervals during the fermentation period and centrifuged at 12,000×*g* for 5 min. The residual raw starch contents of the precipitate were measured by a method described previously [20]. The ethanol fermentation supernatant samples were filtered through a 0.22 μm filter, and the ethanol and released glucose were determined by high-performance liquid chromatography (LC-10AT, Shimadzu, Tokyo, Japan) coupled with a refractive index detector. An Aminex^®^ HPX-87H ion exclusion column (300 × 7.8 mm, Bio-Rad, Hercules, CA, USA) was used to separate the compounds at 60 °C in a 5 mM H_2_SO_4_ mobile phase with a 1 mL/min flow rate. Each analysis was conducted in duplicate. The fermentation efficiency was calculated based on the ethanol yield produced during the fermentation process and the theoretical ethanol yield, following the equation: Fermentation efficiency (%) = produced ethanol (g) × 100/theoretical yield (g), where the theoretical ethanol yield was calculated based on the assumption that 0.567 g of theoretical ethanol yield can be produced from 1 g of starch [69, 70].

### Deletion analysis of the starch-binding domain for raw starch-digesting glucoamylase rPoGA15A

To investigate the effect of the starch-binding domain (SBD) on the activity of the rPoGA15A enzyme towards raw starch, the SBD-deleted *PoGA15A* glucoamylase gene was cloned and heterologously expressed in *P. pastoris* using the methods described above. The expression culture supernatant was obtained by centrifugation after harvest. The recombinant protein was purified using nickel-affinity chromatography and analyzed by SDS-PAGE. The specific activities of the purified enzyme lacking a SBD towards various kinds of starches were determined by comparing it to the wild-type enzyme rPoGA15A under the standard assay conditions mentioned above.

### Nucleotide sequence accession numbers

The nucleotide sequence of the cDNA encoding the RSDG PoGA15A enzyme from *P. oxalicum* GXU20 was submitted to the GenBank database with assigned accession number KX434574.

## Additional files

**Additional file 1: Table S1.** Effects of metal ions and chemicals on the enzyme activity of purified PoGA15A.

**Additional file 2: Figure S1.** Theoretical three-dimensional structure of PoGA15A. The structural model was constructed using SWISS-MODEL software and is based on the known crystal structure of *H. jecorina* glucoamylase (2vn7.1.A).

**Additional file 3: Figure S2.** SDS-PAGE analysis of the recombinant rPoGA15A and native PoGA15A. Lane 1, protein molecular weight marker; lane 2, the purified recombinant rPoGA15A; lane 3, the native PoGA15A.

**Additional file 4: Figure S3.** SDS-PAGE analysis of the rPoGA15A that lacked a SBD and wild-type rPoGA15A. Lane 1, protein molecular weight marker; lane 2, the rPoGA15A lacking SBD; lane 3, the wild-type rPoGA15A.

## Abbreviations

RSDG: raw starch-digesting glucoamylase
SDS-PAGE: sodium dodecyl sulfate-polyacrylamide gel electrophoresis
HPLC: high-performance liquid chromatography
SEM: scanning electron microscopy
BLAST: basic local alignment search tool
PCR: polymerase chain reaction
cDNA: complementary deoxyribonucleic acid
SBD: starch-binding domain
SSF: simultaneous saccharification and fermentation
