# Supplementary material for: Efficient hydrolysis of raw starch and ethanol fermentation: a novel raw starch-digesting glucoamylase from Penicillium oxalicum
Source: Biotechnol Biofuels. 2016 Oct 18;9:216. doi: 10.1186/s13068-016-0636-5 (PMC5069817; doi:10.1186/s13068-016-0636-5)
Supplement: Supplementary file 1 — Additional file 1: Table S1. Effects of metal ions and chemicals on the enzyme activity of purified PoGA15A. [file 13068_2016_636_MOESM1_ESM.docx]

**Additional file 1: Table S1.** Effects of metal ions and chemicals on the enzyme activity of purified PoGA15A.

| Compounds | Concentration (mM) | | Relative activity (%) |
| --- | --- | --- | --- |
| None | - | 100 | |
| K^+^ | 10 | 105.5 ± 0.2 | |
| Na^+^ | 10 | 104.5 ± 2.3 | |
| Mg^2+^ | 10 | 103.9 ± 2.1 | |
| Ca^2+^ | 10 | 101.7 ± 1.5 | |
| Ni^2+^ | 10 | 97.1 ± 1.1 | |
| Zn^2+^ | 10 | 93.1 ± 0.7 | |
| Li^+^ | 10 | 99.2 ± 1.8 | |
| Mn^2+^ | 10 | 127.5 ± 0.4 | |
| Cu^2+^ | 10 | 71.3 ± 3.1 | |
| Fe^2+^ | 10 | 117.2 ± 0.8 | |
| Fe^3+^ | 10 | 93.4 ± 1.2 | |
| Co^2+^ | 10 | 104.9 ± 0.6 | |
| Ag^+^ | 10 | 51.1 ± 1.8 | |
| EDTA | 10 | 100.1 ± 0.2 | |
| SDS | 10 | 17.2 ± 0.3 | |
| Tween 80 | 10 | 107.6 ± 1.1 | |
| Triton X-100 | 10 | 100.7 ± 2.1 | |

The reaction mixture containing 1% raw cassava flour and citrate-phosphate buffer (pH 4.5) was incubated at 40 °C for 30 min and the enzyme activity was measured using the standard method. The enzyme activity determined in absence of any additives was taken 100%. Data are means ± standard deviation from three replicates. The experiment was repeated three times, and similar results were obtained each time.
