# Supplementary material for: Efficient hydrolysis of raw starch and ethanol fermentation: a novel raw starch-digesting glucoamylase from Penicillium oxalicum
Source: Biotechnol Biofuels. 2016 Oct 18;9:216. doi: 10.1186/s13068-016-0636-5 (PMC5069817; doi:10.1186/s13068-016-0636-5)
Supplement: Supplementary file 2 — Additional file 2: Figure S1. Theoretical three-dimensional structure of PoGA15A. The structural model was constructed using SWISS-MODEL software and is based on the known crystal structure of H. jecorina glucoamylase (2vn7.1.A). [file 13068_2016_636_MOESM2_ESM.docx]

**
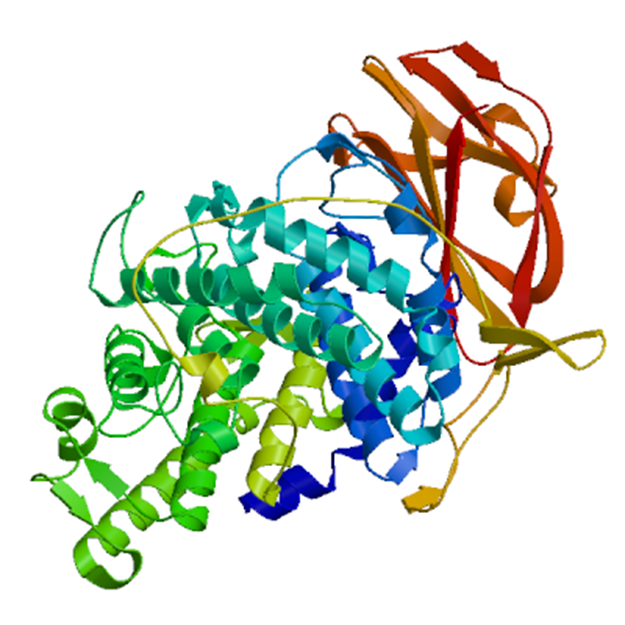
**

**Additional file 2: Figure S1.** Theoretical three-dimensional structure of PoGA15A. The structural model was constructed using SWISS-MODEL software and was based on the known crystal structure of *Hypocrea jecorina* glucoamylase (2vn7.1.A).
