# Supplementary material for: Efficient hydrolysis of raw starch and ethanol fermentation: a novel raw starch-digesting glucoamylase from Penicillium oxalicum
Source: Biotechnol Biofuels. 2016 Oct 18;9:216. doi: 10.1186/s13068-016-0636-5 (PMC5069817; doi:10.1186/s13068-016-0636-5)
Supplement: Supplementary file 4 — Additional file 4: Figure S3. SDS-PAGE analysis of the rPoGA15A that lacked a SBD and wild-type rPoGA15A. Lane 1, protein molecular weight marker; lane 2, the rPoGA15A lacking SBD; lane 3, the wild-type rPoGA15A. [file 13068_2016_636_MOESM4_ESM.docx]

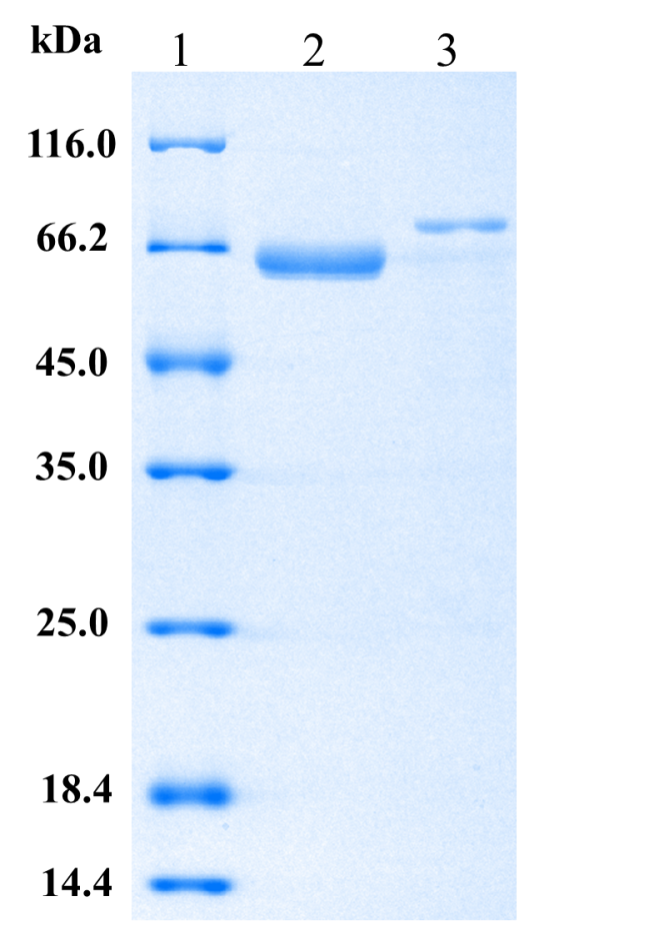


**Additional file 4: Figure S3.** SDS-PAGE analysis of the rPoGA15A that lacked a SBD and wild-type rPoGA15A. Lane 1, protein molecular weight marker; lane 2, the rPoGA15A lacking SBD; lane 3, the wild-type rPoGA15A.
